# Applications of Up-Flow Anaerobic Sludge Blanket (UASB) and Characteristics of Its Microbial Community: A Review of Bibliometric Trend and Recent Findings

**DOI:** 10.3390/ijerph181910326

**Published:** 2021-09-30

**Authors:** Maria Cristina Collivignarelli, Alessandro Abbà, Francesca Maria Caccamo, Silvia Calatroni, Vincenzo Torretta, Ioannis A. Katsoyiannis, Marco Carnevale Miino, Elena Cristina Rada

**Affiliations:** 1 Department of Civil Engineering and Architecture, University of Pavia, Via Ferrata 3, 27100 Pavia, Italy; mcristina.collivignarelli@unipv.it (M.C.C.); francescamaria.caccamo01@universitadipavia.it (F.M.C.); silvia.calatroni01@universitadipavia.it (S.C.); marco.carnevalemiino01@universitadipavia.it (M.C.M.); 2 Interdepartmental Centre for Water Research, University of Pavia, Via Ferrata 3, 27100 Pavia, Italy; 3 Department of Civil, Environmental, Architectural Engineering and Mathematics, University of Brescia, Via Branze 43, 25123 Brescia, Italy; alessandro.abba@unibs.it; 4 Department of Theoretical and Applied Sciences, Insubria University of Varese, Via G.B. Vico 46, 21100 Varese, Italy; vincenzo.torretta@uninsubria.it; 5 Laboratory of Chemical and Environmental Technology, Department of Chemistry, Aristotle University of Thessaloniki, 54124 Thessaloniki, Greece; katsogia@chem.auth.gr

**Keywords:** anammox, microbial community, anaerobic digestion, wastewater, sewage, granular biomass

## Abstract

The interest in research on up-flow anaerobic sludge blanket (UASB) reactors is growing. The meta-analysis of bibliometric data highlighted the growing interest in four diverse topics: (i) energy recovery production; (ii) combination with other treatments; (iii) the study of processes for the removal of specific pollutants and, (iv) characterization of microbial community and granular sludge composition. In particular, the papers published in the first 6 months of 2021 on this process were selected and critically reviewed to highlight and discuss the results, the gaps in the literature and possible ideas for future research. Although the state of research on UASB is to be considered advanced, there are still several points that will be developed in future research such as the consolidation of the results obtained on a semi-industrial or real scale, the use of real matrices instead of synthetic ones and a more in-depth study of the effect of substances such as antibiotics on the microbiota and microbiome of UASB granular biomass. To date, few and conflicting data about the environmental footprint of UASB are available and therefore other studies on this topic are strongly suggested.

## 1. Introduction

The up-flow anaerobic sludge blanket (UASB) systems were first proposed in the 1970s and, recently, the interest in using this technology has grown [1]. In the UASB process, the biomass is not of the flock type but of granular consistency due to a phenomenon in which microorganisms formed granular groups with a more compact structure, a higher dimension, higher density and higher settling capacity than in the conventional active sludge (CAS) [2]. The process is activated using selective environmental conditions that generate a spontaneous involvement of microorganisms commonly present in CAS [3,4]. One of the most significant disadvantages is represented by the low rate of formation and growth of granular biomass that make necessary long start-up periods [5]. However, the inoculation of biomass already granulated proved to be an effective method to reduce the start-up phase [6]. Other drawbacks of this technology include the difficulty associated with the operation of the three-phases separation, the possible sludge washout and foam formation [7,8,9,10].

UASB can be operated in psychrophilic, mesophilic, or thermophilic conditions depending on the type of matrices fed. Generally, higher temperatures allowed researchers to also degrade recalcitrant chemical oxygen demand (COD), particularly in the case of industrial wastewaters (WWs) [1]. The high concentration of biomass (60–100 kg_VSS_ m^−3^) [11] and the high microbial diversity present in the granules allow for the rapid degradation of the organic substance. In compact reactors, the UASB process can also be applied to waste with high organic concentration [12].

One of the main advantages of UASB system is represented by the production of methane and therefore the feasibility of energy recovery. This aspect contributes to the significant widespread of these reactors in low–middle-income countries [13,14]. This is a crucial aspect since water sanitation and energy production are inserted in the Sustainable Development Goals (SDGs) of the United Nations (SDG 6 and SDG 7, respectively) [15]. The other main advantage of this system is represented by the low sludge production with respect to other types of biological treatments [5]. Additionally, this point is a very current aspect since on the one hand legislation about sludge reuse is becoming stricter [16,17] and on the other hand waste (and also sludge) prevention and minimization is strongly stimulated by regulatory bodies (e.g., [18]).

Despite the high organic substances removal efficiencies, the final effluent of a UASB process generally requires subsequent treatments to remove the residual pollutants, particularly nutrients and pathogens [5]. Many studies evaluated the efficiency of diverse post-treatment in order to increase the effluent quality. For instance, de Oliveira and Daniel [19] found 28–33 oocysts L^−1^ of *Cryptosporidium* spp. and 3177–4267 cysts L^−1^ of *Giardia* spp. in UASB effluent and obtained good removal rates (2-log) treating it with dissolved air flotation. In another case, dos Santos and van Haandel [20] used waste stabilization ponds to treat UASB effluent pointed out an acceleration of the decay of pathogens and the removal of nutrients.

This paper aims to review the existing literature on UASB for two different purposes: (i) to analyse bibliometric trends from 1990 to date with a particular focus on those of 2021, and (ii) to present and critically review the results of the first semester of 2021 focusing on the applications of this technology (conventional and combined), on the recent findings concerning the microbiota and the microbiome of the UASB granular biomass and on the environmental footprint of the process. The goal is on the one hand to highlight the hottest issues of UASB on which research is most focused and on the other hand to highlight the gaps in research that still persist. This work therefore aims to be a useful tool for the researcher, suggesting future research ideas based on the results obtained so far, and for UASB plants operators, presenting the most recent results on this topic.

## 2. Methodology

### 2.1. Criteria of Identification, Screening, and Inclusion

In order to develop the following review, scientific peer-reviewed literature was searched and selected according to preferred reporting items for systematic reviews and meta-analysis (PRISMA) guidelines [21].

To consider only peer-reviewed documents, the Scopus^®^ database was used to search relevant literature on this topic. The analysis has been conducted on 5 July 2021, searching the keyword “UASB” on fields “Article title, Abstract, Keywords” using the following query: (TITLE-ABS-KEY (UASB)).

Two diverse screenings were made to obtained diverse groups (G) of data. Criteria of selection were English language, publications on journals, as books or as scientific proceedings for both screenings. Moreover, in screening 1 only literature published from 1990 to 2021 was assessed of eligibility. In the case of screening 2, the period of selection was 1 January 2021 to 5 July 2021.

In the case of screening 1, all records assessed for eligibility were included in G1. Considering that the aim of this review is to analyse very recent applications of UASB and findings on its microbial community, the 109 remaining documents of screening 2 were verified individually to exclude papers that do not treat specifically subject in the scope of the present work (e.g., studies specifically focused on the treatment of UASB effluents) or that do not present new original results (e.g., other review papers). Only remaining works were included in G2.

### 2.2. Analysis of Data

This analysis aims to investigate two different aspects: (i) evaluate the literature trend about UASB technology and applications (based on data selected in G1 and G2), and (ii) present and critically discussed the recent results of literature about the application of UASB, the structure of its microbial community and environmental footprint of the process (based on data selected in G2).

Selected records of both groups were subjected to meta-analyses using VOSviewer software [22,23]. In the case of G1, co-occurrences by author keywords were analysed setting “full counting” mode and limiting visualization only to the set of connected items. Considering that the term UASB may have also been used as an abbreviation in records that deal with issues other than those that are the subject of this review, to avoid this interference, the minimum number of occurrences of each keyword was set to 20.

In the case of G2, the same analysis was developed considering a minimum number of occurrences of each keyword equals 2. Selected records in G2 were also qualitatively and critically analysed to present recent results but also to discuss the current main gaps of literature and possible tips for future research.

## 3. Results

### 3.1. Literature Trend

**Figure 1:**
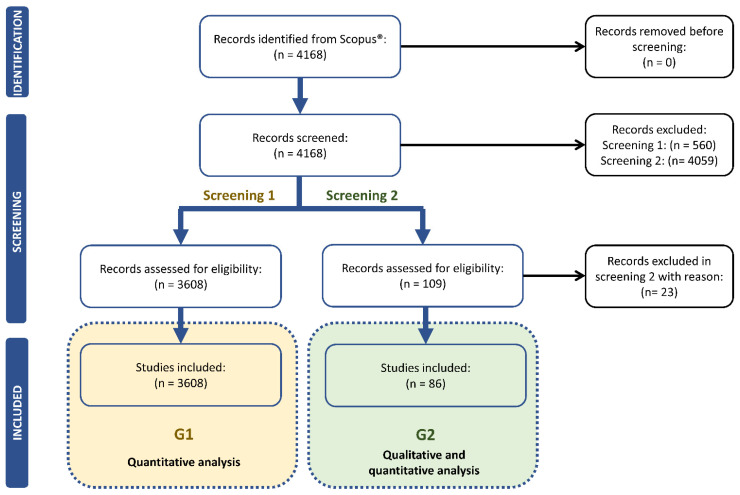
Results of the literature search and studies included in G1 and G2 of the analysis. n: number of records. G: group.

The literature trend on UASB was analyzed selecting two groups of records. 3608 and 86 works were included in G1 and G2, respectively, according to Figure 1.

**Figure 2:**
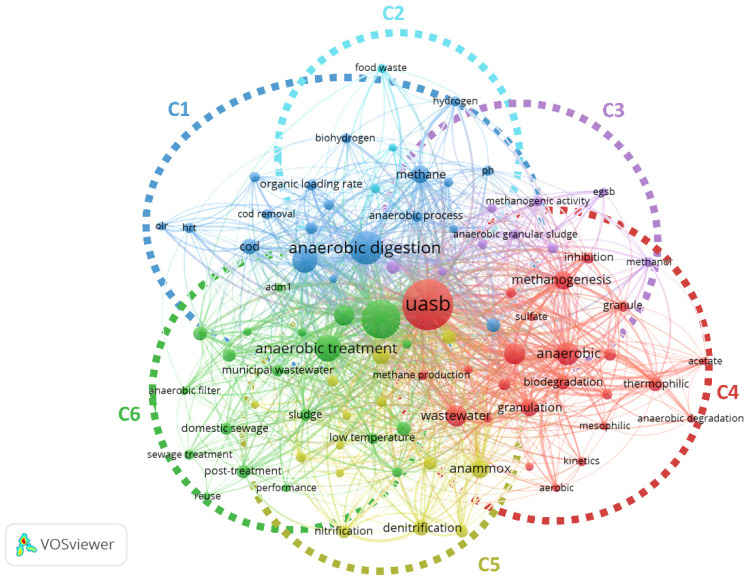
Network visualization of co-occurrences by author keywords of records in G1. Diverse colours represent diverse clusters. Cluster analysis and graphic representation were made using VOSviewer software. C: cluster.

Data in G1 were analysed to evaluate the trend of literature in the last 30 years, finding a very heterogeneous situation. 89 keywords responding to the criteria of the analysis were grouped in six diverse clusters (Figure 2). Excluding the term “UASB”, which was selected as keywords for records identification in Scopus^®^, the terms “anaerobic digestion”, “anaerobic”, “anaerobic treatment”, “biogas”, and “wastewater treatment” were among the most used.

**Figure 3:**
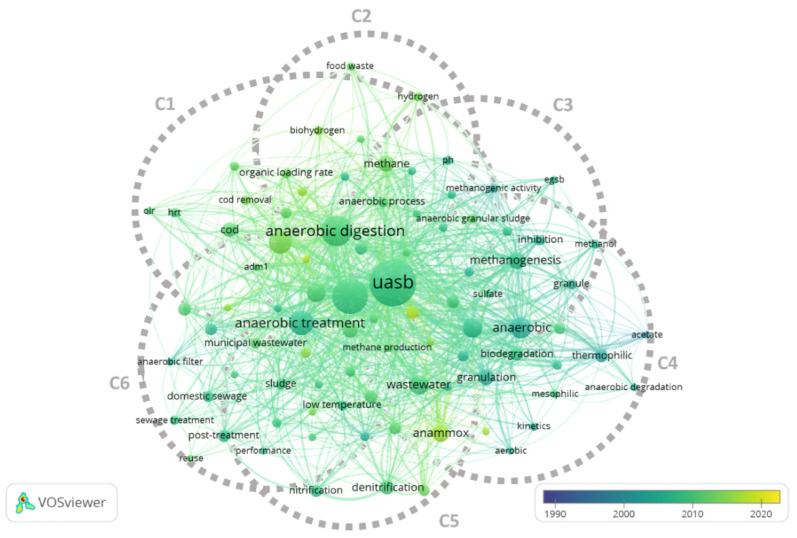
Overlay visualization of co-occurrences by author keywords of records in G1. Diverse colours represent diverse years of publication. Cluster analysis and graphic representation were made using VOSviewer software. C: cluster.

In cluster 1, mainly studies about the optimization of existent reactors (“olr”, “hrt”) to produce methane and biohydrogen are enclosed. These records are strictly interconnected with cluster 2 in which one of the main topics is represented by “food waste”. Considering the age of literature, cluster 1 and cluster 2 include more recent studies especially about energy recovery, specifically biohydrogen and methane production (Figure 3). Cluster 3 and 4 are strictly interconnected and included mainly studies about UASB functioning and granulation activity as the most used keywords are “anaerobic granular sludge”, “egsb”, “methanogenic activity” (cluster 3) and “methanogenesis”, “biodegradation”, “wastewater”, “anaerobic”, “granulation” and “thermophilic” (cluster 4). Based on the present analysis, these keywords are not very current (Figure 3). On the contrary, cluster 5 presents very hot topics keywords such as “anammox” and “microbial community” highlighting a growing interest in processes for nitrogen removal and on studies about microbiota and microbiome of granular sludge. In cluster 6 “anaerobic treatment”, “municipal wastewater”, and “domestic sewage” are among the most used keywords pointing that this cluster mainly includes works on the application of UASB on urban wastewater. In this case, a high number of studies are published between 2000 and 2010. Despite UASB were first proposed about fifty years ago [1], considering all the clusters together (Figure 3), it can be said that before the 2000s the publications on UASB were still limited.

**Figure 4:**
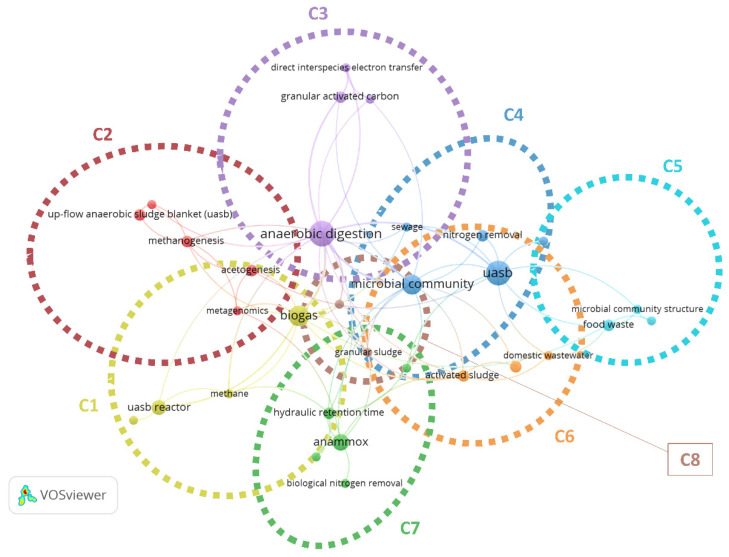
Network visualization of co-occurrences by author keywords of records in G2. A diverse colour represents a diverse cluster. Cluster analysis and graphic representation were made using VOSviewer software. C: cluster.

Data in G2 were analysed to evaluate trend of literature in the last six months. 31 keywords responding to the criteria of the analysis were grouped in eight diverse clusters (Figure 4). Excluding the term “UASB”, the keywords “anaerobic digestion”, “microbial community”, “biogas”, and “anammox” were the most used confirming results of data analysis of G1. To date, the research is focused on four diverse hot topics: (i) methane and biohydrogen production and subsequent energy recovery (C1, and C2); (ii) combination with other treatments (e.g., GAC (C3) or CAS (C6)); (iii) the study of processes for the removal of specific pollutants (e.g., nitrogen removal (C7)); and (iv) characteristics of the microbial community (C4, and C5) and granular sludge composition (C8).

### 3.2. Organic Substance Removal and Biogas Production

To degrade organic substances and produce methane, the UASB process was tested on synthetic matrices [24,25,26,27,28,29] and real feed, mainly toilet wastewater [30,31], urban wastewater [32], distillery wastewater [33], mining wastewater [34,35], leachate [35,36], brewery wastewater [37], food waste [38], soybean molasses [39], pharmaceutical wastewater [40], and pulp mill wastewater [41].

The results of the literature analysis highlighted that UASB used in the research were mainly at lab-scale with few studies conducted at semi-industrial or full-scale reactors (Table 1). The few studies at full-scale concerned the optimization and/or the monitoring of the existing plant. For instance, Omine et al. [33] evaluated the minimum alkalinity supplementation to optimize COD removal from a distillery wastewater. They monitored a real UASB operating in thermophilic conditions (55 °C) for more than one year and they found that 0.045 mg_CaCO3_ mgCOD^−1^ is required to obtain 87% of the COD removal rate. Monitoring two real full-scale UASB reactors, de Freitas Melo et al. [42] studied the seasonality of biogas production finding a negative correlation with the rainfall events.

Lab-scale reactors have been mainly used to investigate the effect of diverse types of matrices on UASB performance but also to simulate critical conditions. For instance, Cervantes-Avilés et al. [43] tested the effect of chronic exposure to titanium dioxide nanoparticles and their accumulation in the granular sludge demonstrating that this aspect did not affect badly the removal of organic substances (92–98%). In their study, an increase in biogas production by 8.8% was evaluated but no significant changes in terms of methane content (78–90%) were detected. Other results of UASB applications in lab-semi-industrial or full-scale reactors are reported in Table 1.

In some cases, UASB technology was also tested to produce volatile fatty acids (VFAs). For instance, Eregowda et al. [44] fed the foul condensate collected by a Kraft paper mill to a UASB reactor operating with HRT equal to 75 h and diverse temperature conditions (22 °C, 37 °C and 55 °C). Their results showed that 52–70% of the organic carbon used (42–46%) was converted into VFAs. Moreover, after more than 5 months of operational activity, Eregowda et al. [44] also found that the biomass of the UASB reactor at 55 °C exhibited the highest activity.

**Table 1:** Several applications of UASB technology for organic substance removal and biogas production. S: Synthetic substrate; R: real matrix. n.a.: not available. ^a^: referred only to soluble COD. ^b^: value deduced by figure analysis. ^c^: tests were performed in batch with granular sludge extracted from a UASB. ^d^: expressed as BOD. ^e^: two reactors in series. ⌀: diameter.

Substrate		Volume of the Reactor (L) [Scale]	Influent COD (mg L^−1^)	Operational Parameters	COD Removal (%)	Biogas and/or Methane Production	Other Results	References
S/R	Type of Real Substrate							
R	Toilet wastewater	2 [Lab]	18,800–33,500	OLR: 0.9–12.4 kg_COD_ m^−3^ d^−1^; T: 52 °C; HRT: 2.5–20 d	77.5–83.6	methane yield: 55.9–60.8%	High phosphorus precipitation	[31]
R	Mining wastewater	1.9 [Lab]	116.9	T: 28.1 °C; HRT: 12.6 d; pH: 6.1	88	n.a.	High heavy metals precipitation	[34]
R	Leachate and carob powder	200 [Sem-ind]	26,100	OLR: 26.1 kg_COD_ m^−3^ d^−1^; T: 35.2 °C; HRT: 1 d; pH: 6.9–7.2; carob powder (⌀: 0.250 mm)	97	Biogas: 2.06 L/Lleachate		[36]
R	Toilet wastewater	1 [Lab]	2000–7000	OLR: 16 kg_COD_ m^−3^ d^−1^; T: 35 °C; HRT: 0.25 d	75.6	methane production potential: 61.9%	The methanogenic activity and the hydrolysis of particulate COD were promoted by the formation of calcium phosphate granular sludge	[30]
S		6 [Lab]	n.a.	OLR: 4.9–7.8 kg_COD_ m^−3^ d^−1^; T: 19.7–25.6 °C; HRT: 30 h; SRT: 82–92.3 d; pH: 6.5; intermittent conditions with 0.5 d (feed period per cycle) and 3 d (feedless period per cycle)	26.8–78.5	methanization of COD: 2.5–37.8%	Contemporary removal of estrogens by biological degradation and adsorption	[24]
R	Ethanol wastewater	4 + 24 ^e^ [Lab]	65,800	OLR: 20–32 kg_COD_ m^−3^ d^−1^; T: 37 °C; pH: 5.5	47–56	Biogas production: up to 67 L d^−1^ (first reactor); up to 300 L d^−1^ (second reactor) ^c^		[45]
S		Lab	9500–16,700	pH: 7–7.9	63.7–82.2	methane production: 0.4–0.6 dm^3^ g_COD reduced_^−1^	The core microbiome was composed of *Methanothrix soehngenii*, *Methanoculleus* sp., unknown *Bacteroidales* and *Spirochaetaceae*	[25]
R	Brewery wastewater	592,000 [Full]	1057–2866	T: 30.6–35.8 °C; HRT: 11 h; pH: 6.3–9.07	71.8–85.5	methane production: 1170.1 Nm^3^ d^−1^		[37]
R	Urban wastewater	2500 [Sem-ind]	95–256	T: 20–25 °C; HRT: 3–5 h; pH: 6.9–7.3	38–85	biogas production: 61–75 L m^−3^ d^−1^		[32]
S		1 [Lab]	10,000	T: 35 °C; OLR: 2.5–10 kg_COD_ m^−3^ d^−1^; HRT: 1–4 d; High salinity conditions (10 gNa^+^ L^−1^); pH: 6.9–7.5	40–80 ^b^	methane production: 0.4–1.73 L L^−1^ d^−1^	Biochar/Fe addition improved the biofilm formation under high salinity conditions allowing the effective treatment of high saline wastewater	[26]
R	Soybean molasses	12 [Lab]	500–4000	HRT: 12–48 h; OLR: 0.25–7 kg_COD_ m^−3^ d^−1^; T: 23–25 °C; pH: 7.6–7.9	70–83	methane production: 23.3–376.2 mL gCOD^−1^; methane content in biogas: 75.5–82.1%		[39]
R	Food waste	6.15 [Lab]	30,000	HRT: 10 d; OLR: 21.9 kg_VS_ m^−3^ d^−1^; T: 36.5–37 °C; pH: 6.8–7.2	88.8	biogas production: 640 L kg food waste^−1^; methane content in biogas: 62.2%		[38]
S		4 [Lab]	3000–6000	HRT: 12 h;OLR: 6–12 kg_COD_ m^−3^ d^−1^; T: 37 °C; pH: 7–8 ^b^	80 42.6 (in shocking load phase)	n.a.	Variation in microbial community after the restoration from shock loading.	[27]
S		3.5 [Lab]	4000	Wastewater with allicin: 12.47 mg L^−1^; T: 30 °C; HRT: 6–24 h; Up-flow velocity: 0.04–0.64; OLR: 4–16 kg_COD_ m^−3^ d^−1^; pH: 7.5–8	74.3–93.3	n.a.		[28]
R	Barley crop residues	0.3 ^c^ [Lab]	168.8	T: 37 °C; pH: 6.85–6.97	n.a.	methane production: 18.02 N mL g_VS_^−1^		[46]
R	Urban and food wastewater	2.2 [Lab]	26,500	MgSO_4_: 150 mg L^−1^ T: 35 °C; intermittent feeding	83.1	methane production: 0.36 L gCODrem^−1^; methane content in biogas: 61.4%	The addition of Mg^2+^ also stimulated the ammonia-nitrogen removal, reducing the inhibitory effect of ammonia on the performance of the process	[47]
R	Pulp mill wastewater	3 [Lab]	2616 ^a^	HRT: 18 h; Flowrate: 4.08 L d^−1^; pH: 7.5–8.5; T: 22 °C	78 ^a^	n.a.		[41]
R	Fish cannery wastewater	n.a.	5992 ^d^	HRT: 33 h	90 ^d^	n.a.		[48]
R	Pharmaceutical wastewater	5.2 [Lab]	1976	OLR: 0.5–2 kg_COD_ m^−3^ d^−1^; HRT: 18 h	49	n.a.		[40]
S		7.8 [Lab]	4300–8300	+chitosan; OLR: 2.1–13 kg_COD_ m^−3^ d^−1^; Up-flow velocity: 0.05–0.15; T: 23.3 °C; pH: 7–7.5	≥70	0.19 Nm^3^CH_4_ kgCOD^−1^ (intermittent feeding); 0.31 Nm^3^CH_4_ kgCOD^−1^ (continuous feeding)		[29]
R	Leachate and mining wastewater	7 [Lab]	2000–2500 ^b^	HRT: 8–30 h; pH: 7.5; T: 35 °C	67–83	methane production: 1600–1800 mL d^−1 b^		[35]

Recently, UASB reactors have also been tested coupled with other technologies such as polishing ponds [49], sponge filters [49,50,51,52], granular activated carbon [53], aerobic treatments [54,55]. Additionally, in this case, diverse matrices were evaluated as possible feed to UASB but very few experiments have been conducted on a larger scale while the majority have been studied in laboratories (Table 2).

The aim of coupling technologies was the improvement of organic substance removal efficiency and methane yields production. For instance, Rahman et al. [56] evaluated the performance of UASB reactor, coagulation–flocculation, and aeration to remove organic substances from wastewater of rubber latex production. They found that this combined system proved to be effective by reducing total Kjeldahl nitrogen (TKN) by 68–87%, and BOD and COD by more than 80% [56].

In another study, Zhang [57] treated oilfield wastewater with a multi-system in which UASB is combined with dissolved air flotation, yeast bioreactor, and biological aerated filter, highlighting organic substance removal equals 96%.

Mazhar et al. [49] compared the performances produced by two combined systems on urban wastewater: (i) UASB + polishing with ponds and (ii) UASB + downflow hanging sponge (DHS) system. The results of their monitoring pointed out the higher removal yields on COD, BOD and TSS feasible with UASB + DHS system (92%, 82%, and 91%, respectively) compared to UASB + polishing ponds (82%, 74%, and 84%, respectively) and predicted the lower operational costs of UASB + DHS with respect to the other combination [49]. Additionally, Asano et al. [51] evaluated the coupling of UASB and DHS, in this case, to treat food wastewater. Further, in this case, their system allowed to obtained high performance in term organic substance conversion into methane: 58% of total COD was removed and 63–87% of soluble COD was converted into methane.

The coupling of UASB reactors with other treatments was also evaluated by Dohdoh et al. [55]. Based on their results, they suggested the combination of hybrid UASB and integrated fixed-film activated sludge (IFAS) as an alternative to conventional UASB + conventional activated sludge (CAS) in treating urban wastewater obtaining about 95% of COD removal after 6 h of HRT [55]. El-Khateeb et al. [58] tested the coupling of UASB reactor with a downflow reactor in which a hanging non-woven fabric made by polyethylene terephthalate (PET) is located. They demonstrated the ability of this system in removing up to 88% and 90% of COD and BOD, respectively. Moreover, their results suggested that coupling these two technologies allowed to reach high removal rates of bacteria (i.e., faecal coliforms and *E.coli*) [58].

Petropoulos et al. [59] compared the performances of two UASB reactors operating in an extreme condition of low temperature (4 °C), with and without an ultrafiltration membrane. They pointed out two interesting aspects: (i) organic substance conversion into methane occurred also in this condition with an HRT equals to 3 days, and (ii) both systems produced comparable results proving that degradation efficiency was not affected by the coupling with an ultrafiltration membrane [59].

The research has also been focused on evaluating solutions for reducing the impact of load shock. In fact, Soh et al. [60] proved that organic load shock can also stimulate the production of soluble microbial products (SMPs) and identified cyclooctasulfur as a potential indicator of reactor performance. Wang et al. [61] tested the effectiveness of biochar against high organic loading shock in up-flow anaerobic sludge blanket (UASB) reactors. They found that the addition of biochar stimulated the development of an enriched microbiota which helped the system to restore quickly maintaining high performances in terms of organic substances removal and methane production in contrast to irreversible acidification which conventional UASB reactors met [61].

Other results of UASB applications in combination with diverse treatments are reported in Table 2.

**Table 2:** Several applications of UASB coupled with other treatment phases for organic substance removal and biogas production. S: Synthetic substrate; R: real matrix. n.a.: not available; TF: Trickling filters; ST: Sedimentation tank; preAC: Pre acidification in continuous-flow stirred reactor; HCPB: hollow centred packed bed; SBTF: Sponge-bed trickling filters; PMR: Photocatalytic membrane reactor; JN: Jet-nozzle; AR: Aerobic reactor; ABF: anaerobic biofilter; BE-UASB: bioelectrochemical UASB; AnMBR: anaerobic membrane bioreactor; GAC: Granular activated carbon; LBR: Leaching bed reactor; DHS: Downflow hanging sponge. ^a^: referred only to soluble COD; ^b^: value deduced by figure analysis; ^c^: value deduced by the comparison of influent and effluent data; ^d^: value reported following equivalence of units of measure; ^e^: in the same reactor; ^f^: five in series; ^g^: for each UASB; ^h^: system composed of a mixed reactor (hydrolysis and acidogenesis) and a UASB reactor (methanogenesis).

Technologies	Substrate		Volume of the UASB Reactor (L) [Scale]	Influent COD (mg L^−1^)	Operational Parameters	COD Removal (%)	Biogas and/or Methane Production	Other Results	References
	S/R	Type of Real Substrate							
UASB + TF + ST	R	Urban wastewater	n.a. [Full]	252	HRT_UASB_: 7.7 h; SRT_UASB_: 40 d; Percolating time_TF_: 20–25 min; HRT_UASB_: 6.1 h	82.9 ^c^	n.a.	High removal of micropollutants	[62]
preAC + UASB	S		6 [Lab]	814–917	OLR: 0.5–8 kg_COD_ m^−3^ d^−1^; T: 35 °C; HRT_UASB_: 1.5–24 h; HRT_TOTAL_: 3–48 h; pH: 5.5 (using HCl)	86.6–95 ^a^	methane production: 0.2–0.3 L g_COD removed_^−1 b^	With pre-acidification phase, the granules performed more superior stability in the microbial community structure	[63]
UASB-HCPB ^e^	R	Palm oil mill wastewater	5 [Lab]	23,100–30,200	T: 55 °C; pH: 6.8–7.2; HRT: 2 d; OLR: 11.55–16.05 kg_COD_ m^−3^ d^−1^	97.5	methane production: 0.26–0.414 L g_CODremoved_^−1 d^; methane content in biogas: 77.8%		[64]
UASB + SBTF	R	Urban wastewater	14,100 [Full]	514	HRT: 8.5 h; pH: 7.1–7.4; T: 25.4–25.5	89	n.a.		[50]
UASB-PMR ^e^	S		3 [Lab]	1054	HRT: 8 h; T: 37 °C; Pore size of membrane: 0.1 µm; Membrane resistance: 6.38 × 10^11^ m^−1^; UV flux: 8 mW cm^−2^	85 (overall) 91 (after acclimatization) 99 (with PMR at 6.4 h HRT)	methane production: 0.30 L g_CODremoval_^−1^	Photocatalytic and photolytic quorum quenching can operate as antifouling also improving the performance of the system.	[65]
JN-UASB ^e^	S		4[Lab]	n.a.	jet nozzle for hydrogen injection; T: 35 °C; Up-flow velocity: 3.18; OLR: 3.8 kg_COD_ m^−3^ d^−1^; pH: 7.5	89.7	methane production: 0.63 L d^−1^ (theoretical production: 0.7 L d^−1^)	The analysis of archaeal community confirmed that the dominant of hydrogenotrophic methanogen increased up to 75.8% in JN-UASB	[66]
UASB ^f^ + AR	R	Hydrothermal liquefaction wastewater	0.4 ^g^ [Lab]	189,000	T: 65 °C (in the first UASB reactor); T: 40 °C (in the other UASB reactors); HRT_UASB_: 20 d; HRT_AR_: 10 d; OLR: 0.5 kg_COD_ m^−3^ d^−1^	97	Biogas production: 10–80% yields (g_COD_ g_CODIN_^−1^) depending on the monitored period		[54]
UASB+ABF	S		50 [Lab]	1262	HRT_UASB_: 1–10 d; HRT_ABF_: 0.25–5 d; OLR_ABF_: 0.03–0.6 kg_COD_ m^−3^ d^−1^ pH_UASB_: 7; Packed media porosity: 57–86%; ABF media: Polyethylene terephthalate; Surface area per unit volume of packed media: 1425–1903 m^2^ m^−3^	80–90 (UASB) 70–94 (ABF)	methane production: 0.15–0.35 L g_CODremoved_^−1^ (in UASB) ^b d^; methane content in biogas: 60–80 % ^b^		[67]
BE-UASB ^e^	R	Tequila vinasses	4.25 [Lab]	105,000	HRT: 1–7 d; Voltage: 0.1–1 V; T: 35 °C; pH: 7	24–45	methane production: 0.274–0.327 NL g_CODremoved_^−1 d^	In BE-UASB, high CO_2_ and H_2_ production were observed	[68]
UASB-GAC ^e^	R	Urban wastewater	4.7 [Lab]	96–260	GAC: (25 g L^−1^); HRT: 8–12 h; T: 19 °C	69–70	methane production: 45–70 mg g_VSS_^−1^ d^−1 b^	GAC showed a protective effect on methanogenic biomass	[53]
mixed reactor+UASB ^h^	R	Baker’s yeast wastewater	0.84 [Lab]	1550–4100	OLR_mixed reactor_: 2.2–13.7 kg_COD_ m^−3^ d^−1^; OLR_UASB_: 2.2–6.58 kg_COD_ m^−3^ d^−1^; pH_mixed reactor_: 4.4–7; pH_UASB_: 5.5–7.9; T: 35 °C	11.7–36	methane production: 0–1.2 L g_CODremoved_^−1^		[69]
LBR+UASB	R	Ensiled corn stover	2 [Lab]	1000–10,000	HRT: 1 d; pH: 8; T: 38 °C; OLR: 1–10 kg_COD_ m^−3^	41.1–100	methane production: 0.114–0.329 L g_CODinfluent_^−1 d^; methane production: 0.066–0.548 L g_VS_^−1 d^	*Methanosaeta* and *Methanobacterium* played synergistic roles with acetogens to effectively convert volatile fatty acids.	[70]
UASB-DHS ^e^	S		10 [Lab]	1000–6000	T: 35 °C; HRT_UASB_: 17–34 h; HRT_DHS_: 10.9–21.8 h; OLR_UASB_: 0.8–8.48 kg_COD_ m^−3^	85.7–87.3	methane production: 0.29 L g_CODremoved_^−1^		[52]

### 3.3. Target Pollutants Removal

Recently, UASB reactors have been studied not only for organic substances degradation and methane production but also for emerging contaminants removal. These contaminants (such as androgens, progestogens, glucocorticoids and steroids) can affect badly human and wildlife health if present in water and wastewater [71].

The occurrence and removal of these pollutants in two swine farms by the UASB system have been widely investigated by Zhang et al. [72]. They pointed out that among diverse treatment processes (e.g., lagoon and sedimentation tank), UASB and conventional anaerobic digester exhibited the higher removal of steroids.

However, the effectiveness of UASB depends mainly on the type of emerging contaminants. In another study, Deng et al. [73] used UASB coupled with Fe-modified GAC, microbubble ozonation and conventional activated sludge (CAS) or membrane biological reactor (MBR) to remove phenolic compounds from a petrochemical industrial wastewater. They proved that bet performance was obtained with Fe-modified GAC + MBR while UASB was not able to remove effectively phenolic compounds [73].

Additionally, nitrogen could represent a problem in UASB effluents. Thanks to the lower sludge production and the not required additional carbon source, the anammox process represents a valid alternative to conventional aerobic treatment for nitrification/denitrification [74]. For this reason, studies concerning the optimization of nitrogen removal by anammox bacteria in anaerobic conditions were developed. For instance, Wang et al. [75] coupled UASB with gel beads in which anammox bacteria were immobilized aiming to optimize the ratio of polyvinyl alcohol and sodium alginate (PVA/SA) gel in beads. They found that highest performance in term of specific anammox activity (0.365 g_N_ g_VSS_^−1^ d^−1^) were achieved with PVA/SA (12%/2%) gel beads [75].

Karasuta et al. [74] evaluated the influence of HRT and pH in anammox reactors and interestingly, they concluded that the varying pH in the effluent not necessarily means a lower nitrogen removal rate while anammox performance is strictly negatively correlated with the HRT of the system.

Considering that anammox bacteria growth is generally slow [76], researchers try to speed up the process of adding carriers. For instance, Lu et al. [77] evaluated the effects on anammox bacteria growth of the addition of GAC and Fe-modified GAC in UASB reactor finding that carriers addition can increase the aggregation of anammox microorganism and strongly reduce the start-up period of the system from 108 d to 94 d (in case of GAC) and to 83 d (in case of Fe-modified GAC).

The necessity of post-treatment UASB effluents has been pointed out by several authors [19,78]. One of the main reasons is the residual presence of microorganisms and chemical pollutants that could be a potential threat to human and wildlife health. Espinosa et al. [79] evaluated the performance of a combined system UASB-high-rate algal ponds (HRAP) to treat urban wastewater demonstrating that UASB exhibited a lower log-removal against *E.coli* and Somatic coliphages (1.09 and 0.4) with respect to HRAP (4.06 and 1.15), respectively. Their study also highlighted a different behaviour of *E.coli* and virus in the UASB: 90% of *E.coli* coming out of the UASB can be found in the sludge while only 10% in the liquid effluent while for viral indicators, the percentages are exactly reversed [79].

However, the effect of the UASB process on microbiological parameters depends mainly on the type of microorganism. Kumar et al. [80] studied the effect of the UASB reactor on SARS-CoV-2 during the recent COVID-19 obtaining the first values concerning the detection and the removal of this virus in UASB reactors and demonstrating that the UASB process was able to reduce SARS-CoV-2 presence up to 1.3 log.

Other cases of UASB investigations on specific pollutants removal are reported in Table 3.

### 3.4. Microbiota and Microbiome in UASB Reactors

To date, research on the UASB system is focusing also on the characterization of the microbial community in granular sludge and its response to diverse substances. Authors agree that microbiota in UASB reactors depends on operational conditions (e.g., temperature) but also on substrate fed. Kuramae et al. [94] focused on the microbial community in UASB reactor fed with blackwater finding a high correlation between the toilet paper and the methanogenic process. They highlighted that the partial degradation of cellulose by Bacteroidetes and Firmicutes present in fed blackwater activated the methanogenesis process allowing the conversion of the cellulose in methane, thanks also to *Chloroflexi* and *Synergistetes* presence in the granular sludge [94].

Yangin-Gomec and Engiz [95] evaluated the effect on the microbial community of propylene glycol in urban wastewater. They proved that a high concentration of this substance can inhibit the biomass and change the microbiota. Specifically, increasing initial glycol concentration, *Firmicutes* replaced Proteobacteria and *Methanoculleus* replaced *Methanocarcina* [95].

The biomass adaptation mechanism in the UASB reactor according to the feed was also highlighted by Gao et al. [96]. In their tests, co-digesting black water and food waste, rather than just blackwater, determined a change in the dominant groups: from the genus *Bacteroides* to *T78*, from the genus *Methanogenium* to *Methanoculleus* and *Methanospirillum* [96]. Li et al. [97] studied the effect of dodecylbenzene sulfonate- and sulphate-containing wastewater highlighting that a low concentration of this surfactant can accelerate the production of methane and, with low COD (SO4^2−^)^−1^ ratio, *Desulfomicrobium* can be identified as the dominant sulphate-reducing bacteria.

Syutsubo et al. [98] studied the case of a psychrophilic UASB reactor fed with wastewater from the electronics industry containing also tetramethylammonium-hydroxide, monoethanolamine, and isopropyl-alcohol. In the acclimatized sludge, they found a higher number of *Methanomethylovorans*-like cells and *Methanosaeta*-like cells at the surface and in the core of the granular sludge. Until the concentration of pollutants remains tolerable, this adaptation allowed UASB sludge to achieve high performance on organic substances [98].

The adaptation of microbiota to the feed was also confirmed by Callejas et al. [99] which evaluated the temporal evolution of the microbial community in a full-scale UASB reactor treating sugarcane vinasse. They noted the progressive reduction in the microbial diversity since the inoculum and the spontaneous selection of a less diverse microbiota to treat organic substances producing methane. In the treatment of vinasse, they also suggested that Firmicutes played a key role due to their abundance [99].

The importance of this phylum was also pointed out for the degradation of citrus peel waste by Camargo et al. [100]. In this case, *Clostridium* (Ph. Firmicutes) and *Escherichia* (Ph. Proteobacteria) were identified as the main microorganism involved in H_2_ and volatile fatty acids production in the UASB reactor [100]. Fang et al. [101] observed that 6 months of continuous feeding with synthetic sewage, and increasing nitrogen concentration, changed the most abundant phyla from Proteobacteria (69.5%), Bacteroidetes (12.1%), and Firmicutes (11.8%) to Proteobacteria (71.7%) and Actinobacteria (16.7%).

In recent years, the combination of the UASB reactor with other processes is gaining attention. Very recently, the impact on the microbial community has also been studied. For instance, Yu et al. [102] studied the effect of the addition of self-fluidized GAC into the UASB reactor. In the GAC-amended reactors, they found that the better performance in terms of methane production with respect to UASB alone can be explained by the enrichment of interspecies electron transfer participants (for instance, *Geobacter* and *Methanosarcina*).

Additionally, the location of GAC can influence microbiota and reactor performances. Yu et al. [103] compared microbial communities of settled and floated GAC finding strong differences. For instance, floated GAG biofilm was primarily constituted by *Methanosarcina* while settled GAC by *Methanobacterium*. These differences in microbiota are reflected in the performance of the system. In fact, their study showed that floated GAC allowed to obtained higher methane production and effluent quality with respect to settled GAC [103].

Xue et al. [104] treated coal gasification wastewater by microelectrolysis-assisted UASB. In this process, a Fe(II)/Fe(III) cycle was triggered by mycroelectolysis with the aim of organic substance and nitrogen removal. In this system, they found that the more efficient removal of pollutants could be related to the spontaneous contemporary presence of iron-oxidant denitrifying microorganisms (*Thiobacillus* spp. and *Acidovorax* spp.) and iron-reducing bacteria (*Geothrix* spp. and *Ignavibacterium* spp.) [104].

Denitrification in UASB sludge and the influence of microbial community were also studied by Carboni et al. [105]. Adding electron donors (such as sulfur, pyrite, thiosulfate, and sulfide), the microbial community diversity significantly reduced and only several genera of autotrophic denitrifiers (such as *Thiobacillus*, *Thioprofundum*, and *Ignavibacterium*) were selected and therefore becoming dominant as reported by [105]. Zhang et al. [106] found that mixotrophic conditions can inhibit the denitrification in the anammox system but at the same time can enhance the ability of *Candidatus Kuenenia* to use diverse types of carbon sources.

Microbial blend to enhance COD removal and methane production performances in bioelectrochemical systems was exploited by Gunaseelan et al. [107]. Mixing anoxygenic photosynthetic bacteria-rich effective microbes with UASB sludge, they obtained higher performances thanks to the synergetic effects of a very heterogeneous microbiota with 28.4% of anoxic photosynthetic bacteria (specifically, *R. palustris* and *R. sphaeroides*) and other microorganisms (71.6%). According to their results, this strengthens the secretion of metabolic intermediates, which stimulate the electron transfer mechanism in the bioelectrochemical system enhancing the performance of the process [107].

Zhang et al. [90] focused their research on the effects of antibiotics and antibiotics resistance genes (ARGs) in swine manure on the microbial diversity of the reactor. They highlighted that biomass was able to effectively remove 82.6% and 71% of 16 diverse antibiotics and ARGs, respectively. However, they found an accumulation of pathogens in the UASB reactor with enrichment of metal resistance genes. Zhang et al. [90] also identify Proteobacteria as the dominant multi-drug resistant microorganism and found *Bacteroides* and *Mycolicibacter* carrying ARGs in untreated swine manure.

He et al. [108] focused their studies on performance recovery by a UASB reactor which was strongly inhibited by oxytetracycline. They highlighted that the organic substance removal and nitrification capacity of the UASB reactor can be easily recovered thanks to the quick rebound of the functional bacteria (e.g., *Mesotoga*, *Longilinea*) once the antibiotic concentration was removed. The researchers also highlighted that the ARG abundance in sludge sampled in the UASB system was lower than in aerobic sludge probably due to the lower horizontal ARGs frequency caused by the lower metabolic activity of anaerobic bacteria [108].

### 3.5. Environmental Footprint of UASB Technology

Several authors tried to assess the environmental impact related to the use of UASB reactors for the treatment of wastewater and sewage sludge. Amaral et al. [109] used the life cycle assessment (LCA) on a sludge treatment plant consisting of a UASB reactor followed by trickling filters, identifying that the biogas produced and subsequently burned was the weak link of the whole system being the main responsible for the emissions of greenhouse gases. Amaral et al. [109] also assessed the impact of the different uses/disposal of the sludge generated in the process by comparing two diverse solutions: (i) reuse in agriculture and (ii) landfill disposal. Their results highlighted that agricultural reuse of sludge achieved a greater environmental impact on the categories of (i) ozone formation, (ii) terrestrial ecotoxicity, (iii) freshwater ecotoxicity, (iv) human toxicity, and (v) terrestrial acidification mainly due to longer transport phase, more sludge treatments, and high heavy metal concentration [109].

However, research has shown conflicting results on this aspect. For instance, Cañote et al. [110] compared three diverse scenarios for the reuse/disposal of biological sludge produced by a UASB reactor: (i) landfill, (ii) further energy recovery, and (iii) reuse in agriculture. Their LCA analysis considered 11 different categories and showed that the recovery option in agriculture is the one that allows for the lowest environmental impact, ensuring the best results in over 70% of the categories studied [110].

The LCA tool was also used by Foglia et al. [111] to determine which plant solution can increase the quality of the effluents for the purpose of irrigation reuse guaranteeing a lower environmental impact at the same time. They evaluated three diverse scenarios, two of which involved the insertion of a polishing treatment with chemical or physical disinfectants to remove the microbial load still present while a third scenario considered the replacement of the CAS with UASB technology followed by an anaerobic membrane reactor. Their results showed that for all categories studied (7 out 8, with the exception of freshwater eutrophication), coupling UASB and anaerobic membrane reactors can guarantee the greatest benefits [111].

## 4. Discussion about Literature Results, Gaps, and Tips for Future Research

The bibliometric review shows that interest in UASB reactors has grown significantly, especially after the 2000s, but has also changed. While in the past, the research topics were mainly based on understanding how the process works, more recently, research has been mainly based on: (i) energy recovery production; (ii) combination of UASB reactors with other treatments; (iii) the study of processes for the removal of specific pollutants (e.g., nitrogen removal); and (iv) characteristics of microbial community and granular sludge composition.

### 4.1. Performances on Organic Substance and Specific Pollutants

Focusing on the literature published in the first semester of 2021, tests on UASB reactors (both single and coupled with other treatments in more complex treatment lines) have alternately focused on two aspects: (i) optimizing the conversion of organic matter into methane for energy production, and (ii) evaluating the removal of specific pollutants such as emerging contaminants and nutrients. The results show that the UASB process can anaerobically treat a wide selection of different types of matrices with different HRTs depending on the type of matrices and the reactor operating temperature. Generally, industrial matrices required a higher HRT (and lower OLR) than urban wastewater due to the greater recalcitrance of the organic substance [32,39,41]. Furthermore, thermophilic reactors have generally allowed researchers to operate in lower HRT conditions thanks to the faster reaction kinetics that permit to fed higher OLR, as has already been confirmed by the previous literature [112]. The methane production was variable and mainly influenced by the process conditions and the matrix used. For instance, Estrada-Arriaga et al. [68] obtained 0.274–0.327 NL_CH4_ g_CODremoved_^−1^ from tequila vinasses using UASB coupled with bioelectrochemical system. Urban and food wastewater has been treated in a mesophilic UASB obtaining 0.36 L_CH4_ g_CODremoved_^−1^ [47].

The application of the UASB process in more complex treatment lines has highlighted the good complementarity with aerobic biological systems and with finishing treatments such as ponds and constructed wetlands. In some cases, the coupling with other technologies had the purpose of obtaining a “hybrid” UASB reactor that could better meet the needs of the case in question. To give some examples, the coupling with media such as GAC for instance to overcome organic overloads [53,61], the upgrading of UASB reactors in bioelectrochemical systems or photocatalytic systems [65,68], the inclusion of sponge bed trickling filters [49,51,52] has all proved to be very promising solutions.

Although the results are to be considered important and can serve as a starting point for future lines of research, some aspects should be highlighted. To date, many of the tests have been conducted on synthetic matrices recreated in the laboratory to simulate water of industrial origin or urban waste. However, this can lead to testing matrices which, although similar, are not comparable in complexity to those of a real nature with results that could be difficult to repeat on real case studies. In future research, therefore, the confirmation of the results using real matrices is to be considered necessary, before a possible large-scale use of plant solutions such as, for example, the coupling of UASB reactors with GAC, the upgrade to bioelectrochemical or photocatalytic systems. It should also be noted that currently most of the research has been conducted on a laboratory scale with reactors of a few L, while the cases of studies on semi-industrial scale plants or full-scale plant monitoring are very limited. On the other hand, the number of research that also takes into consideration the co-digestion of two or more matrices inside UASB reactors is extremely limited. This is an aspect that will certainly have to be further investigated especially in view of a future large-scale application to treat livestock and industrial waste in a more realistic situation. It should be noted that current low-scale tests concern not only the studies on the development of new combined technologies, in this case essentially due to the low maturity of the processes, but also cases of tests with conventional UASBs. Operational and investment costs of these technologies to determine the feasibility of large-scale application of UASB coupled with other technologies represents another aspect in which literature information is almost absent and that should be further investigated.

Given the growing interest in the removal of emerging contaminants, UASB reactors have been tested for the removal of pollutants such as antibiotics, estrogen, pesticides but also nutrients and heavy metals [24,34,72,82,89,92]. The results showed that the process generally guarantees a good coverage of the removal of these pollutants even if there are still some criticalities on the microbial load and on the overall toxicity of the effluent that requires a subsequent finishing treatment [86], as already highlighted previously in the literature [5,112].

### 4.2. Microbiota and Microbiome of Granular Sludge

The study of the microbiota and microbiome of the granular biomass of UASB reactors has been the subject of intense research. Studies have confirmed that the microbiota is able to change its structure to better adapt to external stimuli such as conditions of temperature, feed, and pollutants (e.g., [95,96,97,98,99]). These works could be the starting point for future studies aimed at better exploiting this biomass “adaptation” capacity by optimizing the operation of UASB reactors and increasing their performance. The research interest focused mainly on the possibility of removing nitrogen in anaerobic conditions using anammox processes is attracting increasing interest and for this reason, studies are underway not only to optimize the process within the UASB reactors and but also to better understand the responsible microbiological dynamics.

Studies were also conducted on the effects of antibiotics, which may be present within the treated matrices, on granular biomass and on the responsiveness of UASB systems. UASB systems have not only proved effective in removing antibiotics but also ARGs present in the treated matrix [90,92,108]. This is a very topical issue considering that the resistance of pathogens to antibiotics is now a full-blown problem. The promising results of UASB systems also against these pollutants and ARGs push the need for further studies about the presence, the implications, and the fate of antibiotics in zootechnical slurry but also urban wastewater where the amount of data is very limited.

### 4.3. Environmental Footprint

Some recent studies applied LCA analysis to UASB technology considering the system as a whole or focusing on possible forms of reuse/disposal of the biological granular sludge produced. It should be noted that the results on this issue are still few and sometimes conflicting. Although the results of LCA analysis depend very much on the context in which the technology is inserted, in a case-by-case perspective, it is nevertheless possible to state that the main critical issues highlighted mainly concern (i) the significant emission of greenhouse gases and (ii) the eco-toxicity of the effluent. The first of the two aspects mainly due to the combustion of biogas had already been highlighted in other previous studies [1,113]. The second aspect, on the other hand, is linked to the need for post-treatments, an aspect already highlighted by numerous previous studies [5,112].

Regarding the impact of the reuse of sludge produced by UASB processes in agriculture, the recent LCAs have provided conflicting results [109,110] even if it should be remembered that this result may depend on numerous factors such as, for example, the hypotheses assumed in the realization of the LCA, the typology of matrix treated by the UASB, the operating conditions. Instead, LCAs that compare the solution of the recovery of granular sludge in agriculture with incineration are completely absent but, in the opinion of authors, are strongly suggested.

In fact, it should be noted that the data on this issue are very scarce considering that just three LCA studies concerning UASB implants were published in the first six months of 2021. This aspect is certainly an important gap that should be filled. The authors also suggest evaluating the effect of different post-treatments downstream of the UASB on the results of the LCA, implementing comparative LCAs to identify those processes that best guarantee a reduced impact in environmental terms.

## 5. Conclusions

The meta-analysis of bibliometric data highlighted the change in main aspects of research and the current hot topics: (i) energy production; (ii) combination with other treatments; (iii) the study of processes for the removal of specific pollutants and, (iv) characterization of microbial community and granular sludge composition. UASB proved to effectively remove organic substances with variable yields in terms of methane production. Several applications of UASB in more complex treatment lines are reported in the literature. However, gaps of literature still persist since: (i) many tests have been conducted on synthetic matrices which, although similar, are not comparable in complexity to those of a real nature; (ii) most of the research has been conducted on a laboratory scale reactors while the studies on semi-industrial scale plants or full-scale plant monitoring are very limited; (iii) the research about co-digestion of two or more fed in UASB reactors is extremely limited; (iv) information on operational and investment costs are almost absent. Moreover, studies of the microbiota of the granular biomass confirmed the ability to change its structure to better adapt to external stimuli being the starting point for future studies to optimize the operation of UASB reactors and increasing their performance. Research on the effects of antibiotics proved also that UASB reactors can effectively remove antibiotics and ARGs pushing the need for further studies about the presence, the implications, and the fate of antibiotics in zootechnical slurry but also urban wastewater due to the limited amount of data. Research on UASB environmental footprint gave contrasting results especially concerning the reuse/disposal of excess granular sludge. However, low amounts of data are available so far and other studies on this topic are strongly suggested.

## Figures and Tables

**Table 3:** PCPs: Pharmaceutical and care products; TCE: trichloroethylene; TP: total phosphorus; TF: Trickling filters; Sedimentation tank; PAH: polycyclic aromatic hydrocarbons; PBDE: polybrominated diphenyl ether; ECA-UASB: Electrochemical assisted UASB. AnMBR: anaerobic membrane bioreactor. MPs: microplastics. ^a^: both in aqueous and particulate form. ^b^: removed partially (12.8%) by sulfide-driven partial denitrification and the remaining (87.2%) by anammox. ^c^: value deduced by figure analysis. ^d^: in case of adding anthraquinone-2,6-disulfonate (0–100 μM) and microaeration (0.1 mL min^−1^). ^e^: in case of adding NO_3_^−^ (in a COD (NO_3_^−^)^−1^ ratio of 2.5–10). ^f^: in the same reactor. ^g^: info reported in [81]. ^h^: as sum of oxytetracycline, doxycycline, enrofloxacin, chlorotetracycline, lincomycin, sulfamonomethoxine, ofloxacin, norfloxacin, sulfadiazine, bacitracin, tetracycline, erythromycin-H_2_O, ciprofloxacin, trimethoprim, sulfachlorpyridazine, leucomycin.

Target Pollutant	Technologies	Substrate		Volume of the UASB Reactor (L) [Scale]	Characteristics of the Influent	Operational Parameters	Target Pollutants Removal (%)	References
		S/R	Type of Real Substrate					
Heavy metals	UASB	R	Mining wastewater	1.9 [Lab]	Cu^2+^: 0.18 mg L^−1^ Pb^2+^: 1.72 mg L^−1^ Zn^2+^: 3.07 mg L^−1^ Fe^2+^: 1.76 mg L^−1^	T: 28.1 °C; HRT: 12.6 d; pH: 6.1	Cu^2+^: 70 Pb^2+^: 39 Zn^2+^: 79 Fe^2+^: 65	[34]
Micropollutants	UASB + TF + ST	R	Urban wastewater	n.a. [Full]	triclosan: 67.05 µg L^−1 a^ PAH: 20.19 µg L^−1 a^ estrogen E1: 106.5 ng L^−1^ estrogen E3: 1547.9 ng L^−1^ PBDE: 222.4 ng L^−1 a^	HRT_UASB_: 7.7 h; SRT_UASB_: 40 d; Percolating time_TF_: 20–25 min; HRT_UASB_: 6.1 h	triclosan: 95 ^a^ PAH: 92.2 ^a^ estrogen E1: 88.9 estrogen E3: 99.2 PBDE: 85.6 ^a^	[62]
Nitrate and ammonium	UASB	S		2.0 [Lab]	nitrate: 100 mgN L^−1^ ammonium: 80 mgN L^−1^ sulfide: 20–80 mgN L^−1^	T: 30 °C; HRT: 12 h; pH: 7.8–8.2	total nitrogen: 80 ^b^ sulfide: 100	[82]
Micropollutants	UASB	S		6 [Lab]	estrone: 7000 μg L^−1^; 17α-ethinylestradiol: 3500 μg L^−1^	OLR: 4.9–7.8 kg_COD_ m^−3^ d^−1^; T: 19.7–25.6 °C; HRT: 30 h; SRT: 82–92.3 d; pH: 6.5;intermittent conditions with 0.5 d (feed period per cycle) and 3 d (feedless period per cycle)	estrone: >90 17α-ethinylestradiol: >90	[24]
Heavy metals	UASB	S		1 [Lab]	Pb^2+^: 80–2000 ppm	T: 30–35 °C; HRT: 1 d; pH: 4–7.5 ^c^;	Pb^2+^: 90–100	[83]
PCPs	UASB+MBR	S		20 [Lab]	carbamazepine: 10 μg L^−1^	OLR: 0.1–0.7 kg_COD_ m^−3^ d^−1^; T_UASB_: 29–31 °C; HRT_UASB_: 37 h; SRT_UASB_: >90 d; pH_UASB_: 7–8; T_MBR_: 26–29 °C; HRT_MBR_: 30 h; SRT_MBR_: 90 d; pH_MBR_: 6.8–8.1	carbamazepine: 38.2–48.9 (UASB alone); carbamazepine: 49.8–70 (UASB + MBR)	[84]
Phosphorus	ECA-UASB ^f^	R	Dairy manure	1.2 [Lab]	TP: 264 mg L^−1^	T: 37 °C; Voltages: 0.5–1 V; HRT: 1–12 d ^c^; pH: 7.25–7.75 ^c^	TP: 61.8–65.1	[85]
Toxicity on *D. rerio* and *D. dubia*	UASB	R	Urban wastewater	3 [Lab]	LC_50_ *D. rerio*: 0–30% ^c^ LC_50_ *D. dubia*: 0–10% ^c^	T: 34 °C; HRT: 9 h; OLR: 1.7 kg DQO m^−3^ d^−1^	LC_50_ *D. rerio*: 0–30% ^c^ LC_50_ *D. dubia*: 10–25% ^c^	[86]
Microplastics	UASB+AnMBR	R	Urban wastewater	16 [Lab]	MPs: 3.64 MPs L^−1^	Pore size: 0.03 μm; T: 30 °C; HRT: 6 h	94	[87]
Nitrate and ammonium	UASB	S		4.5 [Lab]	nitrate: 50–200 mgN L^−1^; ammonium: 0–140 mgN L^−1^	T: 25 °C; HRT: 3.8–4.5 h; pH: 7.9–8.7	total nitrogen: >85	[88]
Emerging contaminants	UASB	S		8 ^g^ [Lab]	TCE: 1.46–73 mg L^−1^	T: 35 °C; HRT: 15 h; pH: 7.9–8.7	TCE: >85	[89]
Antibiotics	UASB	R	Swine wastewater	500,000 [Full]	30,000 ng L^−1 h^	HRT: 2.7 d	82.6 ^h^	[90]
Heavy metals and sulphate	UASB	R	Leachate and mining wastewater	7 [Lab]	Mn: 26.80 mg L^−1^ Zn: 0.47 mg L^−1^ Ca: 410.57 mg L^−1^ Mg: 139.85 mg L^−1^ Fe: 1.25 mg L^−1^ Sulphate: 700–900 mg L^−1 c^	HRT: 8–30 h; pH: 7.5; T: 35 °C	Mn: 93.3 Zn: 99.3 Ca: 54.5 Mg: 41.9 Fe: 95.1 Sulphate: 66–78	[35]
Nitrates	UASB	S		1.4 [Lab]	Nitrates: 50 mgN L^−1^	T: 26–33 °C; HRT: 12 h; COD (N-NO_3_^−^)^−1^ ratio: 2–8	Nitrates: 30–100 ^c^	[91]
Antibiotics	UASB	S		3.5 [Lab]	Sulfamethoxazole: 194–219 μg L^−1^; ^d^ Trimethoprim: 198–216 μg L^−1^; ^d^ Sulfamethoxazole: 194–213 μg L^−1^; ^e^ Trimethoprim: 202–215 μg L^−1^; ^e^	HRT: 7.4 h; T: 28 °C; pH: 7	Sulfamethoxazole: 6.2–77.1; ^d^ Trimethoprim: 6.2–91.1; ^d^ Sulfamethoxazole: 11.8–85.8; ^e^ Trimethoprim: 13–86.2; ^e^	[92]
Nitrite and ammonium	UASB	S		0.3 [Lab]	nitrite: 70–150 mgN L^−1^; ammonium: 70–150 mgN L^−1^	HRT: 12–24 h; T: 23–28 °C	Total nitrogen: 55–85	[93]

## Data Availability

All the data available has been presented in this manuscript.
